# Pre-treatment of canine plasma with heat, rather than acid, efficiently enhances *Dirofilaria immitis* antigen detection

**DOI:** 10.1186/s13071-023-06083-7

**Published:** 2023-12-19

**Authors:** Daniel Felipe Barrantes Murillo, Chengming Wang

**Affiliations:** Department of Pathobiology, College of Veterinary Medicine, Auburn University, Auburn, AL USA

**Keywords:** *Dirofilaria immitis*, Immune complex dissociation, Heat treatment, Acid treatment, Heartworm

## Abstract

**Background:**

The dissociation of antigen-antibody complexes has been utilized to enhance the accuracy of serological tests for infectious diseases, including *Dirofilaria immitis*. Currently, the antigen detected by available tests is primarily a glycoprotein found in the reproductive tract of female worms. However, this antigen can become inaccessible when bound to excessive circulating antibodies, leading to reduced test sensitivity and false-negative results. Acid and heat treatments of the sera or plasma have been established as reliable methods for inducing immune complex dissociation (ICD). Previous antigen testing for heartworm infection in dogs and cats has demonstrated that these treatments improve the diagnostic sensitivity without compromising specificity. This study aims to evaluate the performance of four distinct ICD methods in the detection of *D. immitis* antigen.

**Methods:**

We utilized twofold serial dilutions of a well-characterized plasma (ranging from 1:2 to 1:4096) obtained from a *D. immitis*-infected dog to simulate the diverse antigen levels encountered in real-life infected dogs. The presence of antigen in the diluted samples, both without treatment and treated with four ICD protocols, was assessed in triplicate visually using DiroCHEK^®^ by observing color changes. OD values were also obtained using the microplate reader SpectraMax® i Series-Spectramax Id3. A Factorial ANOVA test was conducted to compare the OD values between samples with and without treatments.

**Results:**

The highest dilution at which color changes were observed was 1:128 for untreated samples and for samples subjected to acid treatments in ICD-3 and the hybrid ICD-4 protocol. In contrast, both heat treatment protocols (ICD-1 and ICD-2) exhibited color changes at a 512-fold dilution. The OD values in samples subjected to heat treatment were significantly higher than those in untreated samples, up to dilutions of 512-fold. Although OD values tended to be higher in samples subjected to acid treatment and the hybrid protocol compared to untreated samples up to a 128-fold dilution, this difference was not significant as the samples underwent further dilution.

**Conclusions:**

Our findings affirm that heat treatments, rather than acid treatment, efficiently enhance the detection of *D. immitis* antigen by liberating the sequestered antigen from the immune complexes.

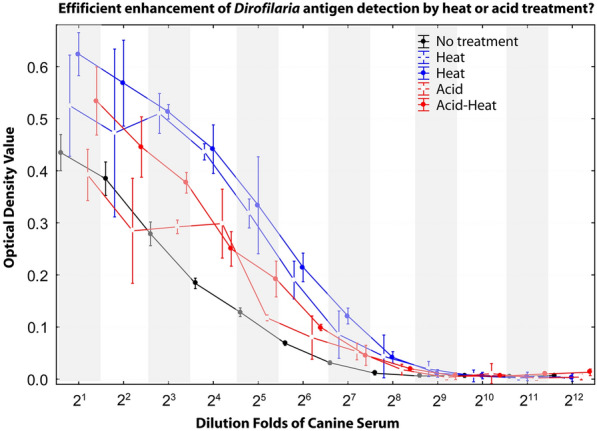

## Background

Heartworm disease is an arthropod-borne illness caused by *Dirofilaria immitis*, a filaroid nematode belonging to the family Onchocercidae [1, 2]. *Dirofilaria immitis* infections are documented in both dogs and cats and exhibit a widespread distribution across the USA [2, 3]. Dogs serve as the definitive hosts, and infections can range from subclinical to severe, presenting as life-threatening clinical conditions marked by respiratory distress, epistaxis, hemoptysis, ascites, exercise intolerance and anorexia [1, 2]. Diagnosis encompasses various methods, including the identification of microfilaria in peripheral blood, molecular detection through PCR and antigen detection—the latter being regarded as the gold standard for heartworm diagnosis [1, 4]. Despite the widespread use of preventatives and educational campaigns, the Companion Animal Parasite Council (CAPC) reported over 200,000 positive canine antigen tests in 2021, underscoring the ongoing prevalence of heartworm infections [5, 6].

Antibody-antigen reactions occur between free antigens in the serum or antigens on erythrocytes and the antibodies present in the serum or plasma [7]. These interactions in antigen-antibody complexes are reversible and can be influenced by various factors, including the distance between the reactive sites of antibodies, the location and number of antigenic determinants, the specificity of the antibody binding site and factors such as time, temperature, pH, ionic strength and the concentration of antigens and antibodies [7].

Dissociation of antigen-antibody complexes has been employed to enhance the accuracy of serological tests for infectious diseases, including *D. immitis*, hepatitis C virus, *Histoplasma* spp., human immunodeficiency virus, dengue virus and *Leishmania infantum* [8–13].

Immune-complex dissociation (ICD) techniques for heartworm diagnosis have been in use since 1985 [14]. However, the dissociation step has been eliminated because of the increased sensitivity of commercially available kits for antigen detection [15, 16]. Currently, the antigen detected by these tests is a glycoprotein predominantly found in the reproductive tract of female worms [17]. This antigen is present in a free state within the plasma or serum but may become unavailable when bound to excessive circulating antibodies, thus reducing the sensitivity of the tests and leading to false-negative results [18].

The purpose of this study is to evaluate the performance of commonly used ICD methods available in the literature to detect the *D. immitis* antigen. We selected two distinct heat-based ICD protocols published by Weil et al., 1985 (ICD-1), and Swartzentruber et al., 2009 (ICD-2), which are widely cited in the literature. Prior to 1995, manufacturers recommended heat treatment before applying antigen tests for ICD [8, 14, 19, 20]. Heat treatment at 104 °C has been reported as a reliable method for ICD, enhancing sensitivity in previous antigen testing for heartworms in dogs and cats without compromising specificity [14]. Heat treatment is also unlikely to produce false-positive results because of cross-reactivity with other intestinal helminths such as *Acanthocheilonema reconditum* and protozoa including *Giardia* sp., *Sarcocystis* sp. and *Cystoisospora* sp. [14].

A third protocol (ICD-3) used in this study was based on acid treatment. This acid treatment ICD protocol, when applied before antigen testing in samples, has been reported to enable the detection of *D. immitis* antigens without inducing false positives [21].

Lastly, we proposed a combination of both methods, using heat and acid, for comparison against the previously described ICD protocols. However, this combined approach has not been described as a diagnostic method for heartworm disease. Through an extensive literature review, we selected a fourth protocol (ICD-4), a hybrid approach that involves an acid solution followed by heat incubation. This protocol was originally designed for HIV antigen detection [10], and its performance for heartworm antigen detection has never been explored.

In this study, we employed twofold serial dilutions of a well-characterized positive plasma with various treatments as well as a control group without any treatment. These dilutions were utilized to mimic different concentrations of *D. immitis* antigen, resembling the diverse antigen levels that may be encountered in the real-life context of infected dogs.

## Methods

### Canine plasma samples

A plasma sample from a *D. immitis*-infected dog served as a positive control in this study. These plasma samples were generously provided by the Filariasis Research Reagent Resource Center, Department of Infectious Diseases, College of Veterinary Medicine, University of Georgia, USA. The dog was infected on March 30, 2021, via inguinal subcutaneous injection of 50 infective third-stage larvae of the Georgia-2 strain of *D. immitis*, following a previously published inoculation protocol [22]. The microfilaremia status was confirmed through microfilaria counts using Giemsa stain on thick blood smears, with an estimated count of 43,416 microfilariae/ml blood, in accordance with established procedures [22]. The plasma was tested to be *D. immitis* antigen positive using a commercially available well-based ELISA (DiroCHEK^®^, Zoetis) for antigen detection.

In addition, a commercially available sterile canine plasma (Innovative Research, Lot 40447) obtained from a specific-pathogen-free dog was employed as a diluent to serially dilute the *D. immitis*-positive sera. Verification of its negative status was accomplished through testing with a commercially available well-based ELISA (DiroCHEK^®^, Zoetis) designed for antigen detection.

### Two-fold dilutions of canine sera, ICD assays and antigen testing

In a sterile 12 ml conical centrifuge tube, 3 ml positive plasma and 3 ml negative plasma were mixed in a 1:1 ratio and incubated at 37 °C for 1 h. Following incubation, the mixture was subsequently diluted into negative plasma through twofold serial dilutions, ultimately reaching a 1:4096 dilution (2^12^). Each dilution was then aliquoted into individual tubes and subjected to *D. immitis* antigen detection, with and without prior treatment of the sera.

After an extensive literature review, two ICD protocols with heat treatment (ICD-1, ICD-2) and one acid treatment (ICD-3) and one acid and heat treatment combination (ICD-4) were included in this study (Table 1) [8, 10, 12, 21].

**Table 1:** Summary of the methodology from each of the four ICD protocols used in this experiment

	Summary of the protocol	References
ICD-1	Mix 100 µl of serum or plasma with 100 µl 0.1 M disodium EDTA (pH 7.5) (1:1 ratio) and incubate at 100 °C for 5 min, followed by centrifugation at 16000×*g* for 5 min	Weil et al. [8]
ICD-2	Mix 600 μl serum or plasma sample with 200 μl 0.1 M disodium EDTA (pH 7.5) (ratio 1:3) and incubate at 104 °C for 10 min. Then, centrifugate at 16000×*g* for 5 min	Swartzentruber et al. [12]
ICD-3	Mix 100 µl of serum with 100 µl of 7.5% (w/v) TCA (1:1 ratio) to achieve pH = 1. Incubate at room temperature for 20 min followed by centrifugation at 16000×*g* for 5 min. Approximately 80% (170 μl) of the total starting volume of serum or plasma and TCA is recovered. Mix 150 μl aliquot of the centrifugated sample + 30 μl 1 M Trizma buffer (in a volume equal to 20% of the volume of the recovered supernatant) and then invert several times to mix, thus returning the sample to a neutral pH (pH = 7–8)	Starkey et al. [21]
ICD-4	Mix 100 μl serum or plasma sample + 300 μl SDS 7 mM; DTPA, 1.5 mM (pH 7.2) solution (1:3 ratio) vortex and incubate at 95–98 °C for 4 min	Steindl et al. [10]

In brief, ICD-1 is a heat-based treatment method that involves mixing the serological sample in a 1:1 ratio with 0.1 M disodium EDTA at pH 7.5. The mixture is then incubated at 100 °C for 5 min, followed by centrifugation at 16000×*g* for 5 min.

Similarly, the ICD-2 protocol, also heat-based, entails combining the serological sample in a 3:1 ratio with 0.1 M disodium EDTA at pH 7.5. This mixture is incubated for 10 min at 104 °C, followed by the same centrifugation procedure as in ICD-1.

ICD-3 is an acid-based protocol, where the serum or plasma sample is mixed with 7.5% (w/v) TCA in a 1:1 ratio to lower the pH to 1. The mixture is incubated at room temperature for 20 min, followed by centrifugation, as in the case of ICD-1. Approximately 80% of the supernatant is recovered and then combined with 1 M Trizma buffer, in an amount equal to 20% of the measured supernatant volume, to restore the sample to a neutral pH (pH = 7–8).

ICD-4 is an acid-heat-based method, in which the serum or plasma samples are mixed with a solution containing 7 mM SDS and 1.5 mM DTPA at pH 7.2 in a 1:3 ratio. This mixture is then incubated at 95–98 °C for 4 min.

Each diluted sample (from 2^1^ to 2^12^) received four treatments as described in Table 1. Subsequently, a well-based ELISA (DiroCHEK^®^, Zoetis) for antigen detection was conducted on each diluted sample without treatment and with four different types of treatments. Each sample was analyzed in triplicate, and one positive and three negative controls were included on each plate.

The interpretation of the antigen testing was conducted visually to determine the presence of antigen or non-detectable antigen (NDA) by observing a color change on the DiroCHEK^®^ as indicated in the manufacturer’s instructions. Additionally, a spectrophotometric OD reading at a wavelength of 415 nm was obtained for each sample using the microplate reader SpectraMax® i Series—Spectramax Id3.

### Statistical analysis

All statistical analyses were performed with the IBM® SPSS® Statistics software package (International Business Machines Corp., Amonk, NY, USA). A factorial ANOVA test was performed to compare the OD values between the samples with and without treatment. A difference at *P* ≤ 0.05 was considered statistically significant.

## Results

During the visual interpretation of antigen testing, a color change on the DiroCHEK^®^ was observed when the *D. immitis* antigen was present in the sample. The highest dilution for which color changes were detected was 1:128 dilution (2^7^) for the untreated samples and for the samples subjected to acid treatments in ICD-3 and hybrid ICD-4 (Table 2). In contrast, both heat treatment protocols (ICD-1 and ICD-2) exhibited a four-fold enhancement in antigen detection, with color changes observed in samples diluted up to 512-fold (2^9^) (Table 2).

**Table 2:** OD values from twofold serial dilutions of positive plasma sample (control) and four ICD protocols

Dilution	No treatment		ICD-1			ICD-2			ICD-3			ICD-4		
	OD ± SD	Color	OD ± SD	P***	Color	OD ± SD	*P*	Color	OD ± SD	*P*	Color	OD ± SD	*P*	Color
2^1^	0.435 ± 0.066*	+ **	0.525 ± 0.039	**0.02**	+	0.624 ± 0.017	**10**^**−4**^	+	0.392 ± 0.020	0.22	+	0.534 ± 0.026	**0.01**	+
2^2^	0.385 ± 0.063	+	0.472 ± 0.065	**0.02**	+	0.568 ± 0.033	**10**^**−4**^	+	0.285 ± 0.041	**0.01**	+	0.446 ± 0.023	0.07	+
2^3^	0.279 ± 0.021	+	0.510 ± 0.015	**10**^**−4**^	+	0.513 ± 0.006	**10**^**−4**^	+	0.292 ± 0.005	0.54	+	0.377 ± 0.008	**10**^**−3**^	+
2^4^	0.184 ± 0.004	+	0.436 ± 0.006	**10**^**−4**^	+	0.441 ± 0.027	**10**^**−4**^	+	0.299 ± 0.019	**10**^**−4**^	+	0.250 ± 0.013	**10**^**−4**^	+
2^5^	0.128 ± 0.013	+	0.318 ± 0.011	**10**^**−4**^	+	0.334 ± 0.002	**10**^**−4**^	+	0.118 ± 0.037	0.19	+	0.192 ± 0.014	**10**^**−4**^	+
2^6^	0.069 ± 0.002	+	0.190 ± 0.015	**10**^**−4**^	+	0.215 ± 0.011	**10**^**−4**^	+	0.080 ± 0.017	0.07	+	0.099 ± 0.002	**10**^**−4**^	+
2^7^	0.031 ± 0.003	+	0.085 ± 0.018	**10**^**−4**^	+	0.121 ± 0.006	**10**^**−4**^	+	0.053 ± 0.006	**10**^**−3**^	+	0.046 ± 0.008	**10**^**−2**^	+
2^8^	0.011 ± 0.005	−	0.044 ± 0.017	**10**^**−4**^	+	0.042 ± 0.004	**10**^**−4**^	+	0.018 ± 0.004	0.06	−	0.020 ± 0.001	**0.02**	−
2^9^	0.006 ± 0.002	−	0.019 ± 0.006	**10**^**−3**^	+	0.011 ± 0.003	**0.04**	+	0.003 ± 0.002	0.27	−	0.006 ± 0.003	0.94	−
2^10^	0.007 ± 0.002	−	0.006 ± 0.005	0.83	−	0.007 ± 0.003	0.98	−	0.011 ± 0.008	0.37	−	0.006 ± 0.000	0.73	−
2^11^	0.004 ± 0.002	−	0.002 ± 0.002	0.22	−	0.005 ± 0.003	0.58	−	0.002 ± 0.002	0.14	−	0.010 ± 0.001	**10**^**−2**^	−
2^12^	0.006 ± 0.002	−	0.004 ± 0.001	0.45	−	0.003 ± 0.003	0.28	−	0.003 ± 0.002	0.37	−	0.013 ± 0.003	0.09	−

^*^The optical density values are expressed as mean ± standard deviation (SD)

^**^The color change based on the DiroCheck is read as + (positive) or – (negative)

^***^P value was calculated by comparing the OD values between NO Treatment group and ICD-1, ICD-2, ICD-3, or ICD-4

**Fig. 1:**
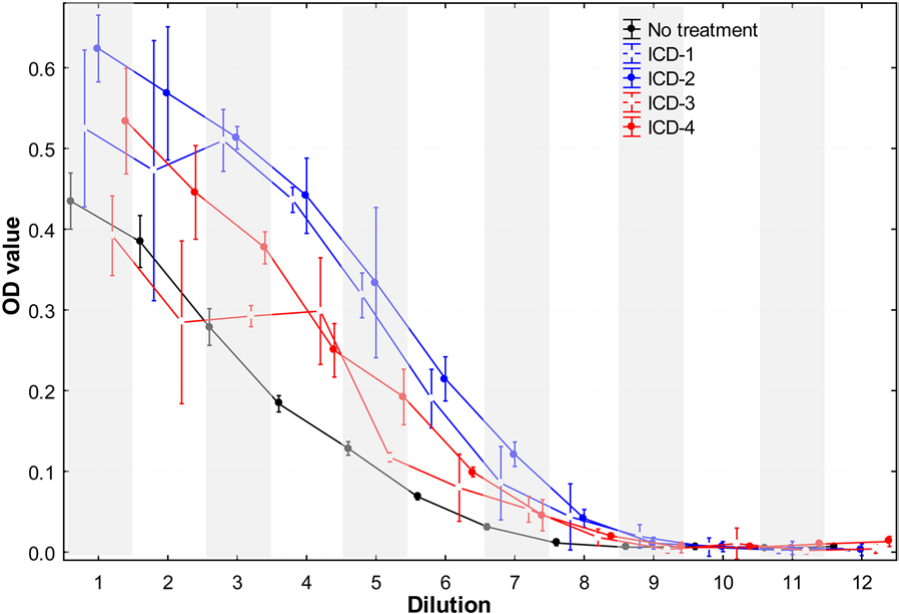
Detection of *Dirofilaria immitis* antigen by ELISA for twofold-diluted plasma samples with and without treatment. A spectrophotometric OD reading at a wavelength of 415 nm was obtained for each sample using the microplate reader SpectraMax® i Series-Spectramax Id3. Filled black circle, untreated sera. Open blue circle, ICD-1 protocol. Filled blue circle, ICD-2 protocol. Open red circle, ICD-3 protocol. Filled red circle, ICD-4 protocol. The error was represented as mean ± 95% confidence interval

A statistical comparison of OD values between samples with and without treatment yielded similar findings. The OD values in samples subjected to heat treatment were significantly higher than those in untreated samples, up to dilutions of 512-fold (2^9^) (Fig. 1, Table 2). While OD values tended to be higher in samples subjected to acid treatment and hybrid protocol compared to untreated samples up to 128-fold dilutions, the difference did not reach statistical significance as the samples were further diluted. Interestingly, at a dilution of 2^11^, samples subjected to ICD-4 treatment displayed significantly higher OD values than untreated samples. This observation may be attributed to the variations from extremely low antigen levels in these samples, as both sets of samples tested negative based on the color change.

## Discussion

Several experimental assays have established that heartworm antigens are widely distributed within the female worm's uterus; however, they can become sequestered within immune complexes [8]. In infected dogs, the antigen may go undetected because of factors present in the blood [23]. Various methods have been tested to release antigens from these complexes without destroying them [8].

This comparative approach, involving the examination of various ICD protocols employing heat and acid treatments with serially diluted samples, has not been previously explored through quantitative measurements. Previous studies validated ICD protocols using convenience samples with unknown concentration of the heartworm antigen. Despite the existing publications that have compared various ICD protocols, our approach stands out as innovative. This is primarily because we directly compare heat and acid protocols, and our use of serial dilutions replicates the natural conditions within the host. In the host, the availability of the antigen decreases because of antigen-antibody interactions, and our approach mirrors this phenomenon. Additionally, we employ OD measurements, which enable a quantitative analysis of the performance of each ICD method, especially when the threshold of undetected antigen is reached.

This study aims to assess the impact of ICD protocols on a positive sample diluted in a negative control, affecting the availability of heartworm antigen. Experimental studies that mimicked antigen blocking by circulating antibodies from non-infected dogs yielded positive results after heat treatment [8, 23]. A diluted standard positive in 50% canine serum allowed a recovery of 100% of the antigen after heat treatment [8]. The antigen was similarly detected when diluted in PBS, 5% FCS or dog serum at a concentration of 50% [8]. Another experimental assay assessed antigen blocking by mixing serum from a positive dog with hyperglobulinemic serum from a negative dog infected with *Hepatozoon americanum*, resulting in complete blocking of the antigen at the 1:16 dilution [23]. Heat treatment applied to these samples retrieved the antigen after complete blocking [23]. These previous studies, however, tested only a limited number of dilutions until complete blocking was reached, without applying heat treatment to all of them. They did not specify the OD values from each dilution after treatment, making a qualitative analysis of the ICD protocols challenging. Furthermore, they did not compare these methods with other ICD techniques.

In a recent study conducted by Gruntmeir et al. in 2023, the researchers assessed antigen detection in mixed antigen-positive heartworm serum using antibody solutions after subjecting them to heat treatment [24]. They measured the OD values employing a commercial heartworm antigen ELISA and conducted protein quantification as well [24]. The authors observed substantial alterations in protein levels and antigen availability when the solutions were subjected to temperatures > 65 °C [24]. These findings strongly support the notion that heat-based methods primarily rely on the denaturation of antibodies as the underlying mechanism. It is important to note that the authors in this study did not undertake a comparison involving acid-based ICD protocols, nor did they employ multiple dilutions.

In another study conducted by Beall et al. in 2016, several heat treatments were compared, including enzymatic treatment (pepsin-based) and acid treatments [18]. The authors concluded that all ICD protocols performed effectively without inducing false-positive results [18]. However, the OD values of the four protocols were measured only in five samples, lacking consecutive measurements or dilutions for making a statistical comparison between the methods. Furthermore, no serial dilutions were conducted using negative plasma, and quantitative measurements were not utilized to facilitate a comprehensive comparison between the different methods.

In this study, our data ambiguously demonstrated that heat treatments can retrieve antigens no longer detectable with the ELISA commercial kit in non-treated samples. Additionally, heat treatment consistently increased the OD value compared to the controls, which is not evident in the acid treatment protocols tested in this study.

Although heat treatment is not routinely recommended, it is performed in animals with multiple antigen tests yielding contradictory results, in cases where there is no antigen detected despite evidence of microfilaria or when clinical symptoms support heartworm infection [25–27]. Recently, heat-based ICD has demonstrated its effectiveness in detecting exclusively male heartworm infections in dogs [28]. In practical terms, among the two heat ICD protocols tested in this study, the protocol from Weil et al., 1985, has the advantage of requiring a smaller sample volume, only 100 µl, in contrast to the Swartzentruber et al. (2009) protocol, which requires a total of 600 µl. This significant difference in sample volume is crucial in cases where the available volume is limited. Both assays, however, share the limitation of requiring serum or plasma dilution in EDTA, which can reduce the proportion of antigen available for testing. A large volume will be required to avoid using EDTA when testing heat ICD.

Acid treatment has been established as a reliable method for detecting *D. immitis* without generating false-positive results in canine serum samples [21]. However, in the case of serum samples from cats, acid treatment has displayed inconsistent results [29]. Compared to heat treatment, there was a slight difference between the outcomes of heat and acid treatment, although this difference did not reach statistical significance [21]. Notably, ICD with acid treatment led to an increase in the OD values of the samples, although not to the same extent as observed with samples subjected to heat treatment [21].

The disparities between our findings and those of other published studies [18, 21] could potentially be attributed to the varying levels of antigen availability in the samples. Previous studies did not investigate serially diluted samples in acid treatment protocols. Earlier experiments reported the effectiveness of acid treatment because of the increased availability of the antigen brought about by pH changes in the samples, even though the OD values were lower compared to heat-treatment protocols [18, 21]. However, the gradual reduction of antigen concentration resulting from serial dilutions was not assessed in these assays, rendering them unable to determine the point at which antigen detection is no longer possible compared to heat-based treatments. ICD-3 offers advantages over the heat treatments, primarily due to its lower sample volume requirement, as little as 50 µl, and the absence of the need for a heat block [21].

An important study by Venco et al. suggested that heat treatment may lead to false-positive results in heartworm antigen testing in ex vivo parasites and in dogs naturally infected with *Dirofilaria repens* and *Angiostrongylus vasorum* [17]. This study, however, possesses several limitations attributed to its highly technical nature. Notably, the positive control samples originate from a singular experimentally infected dog, with no inclusion of naturally infected animals. This stands in contrast to prior publications that utilized samples from naturally infected dogs sourced from animal shelters, where infection was confirmed through necropsy [14, 24, 28]. Another noteworthy limitation is the lack of blood samples from dogs infected with *D. repens* and *A. vasorum*. Such samples could have been instrumental in exploring the potential for false positives induced by heat treatment. However, the conclusion in this study that heat treatment enhances *D. immitis* antigen detection remains robust, as demonstrated by the serial dilution of serum from *D. immitis*-infected dogs with sera from SPF dogs.

Notably, a nationwide molecular survey in companion dogs and cats in the USA did not identify *D. repens* in dogs and cats (0/2334) [1]. *Angiostrongylus vasorum* has been identified in various regions, including the UK, Asia, Africa and Canada, but has not yet been reported in the USA [30–32]. Nevertheless, future research should involve the use of sera from dogs infected with *D. immitis*, *D. repens*, *A. vasorum* and other helminths of interest to systematically assess the impact of heat treatment on antigen detection, as co-infections of these parasites are prevalent in many regions worldwide.

To our knowledge, the ICD-4 protocol has not been previously tested in heartworm research. It consistently increases the OD value compared to non-treated controls. Unlike the acid protocol, it requires only 100 µl and does not require centrifugation. Thus, it could serve as another potential protocol for heartworm diagnosis. However, it is worth noting that this protocol was unable to retrieve antigens after reaching the threshold of non-detectable antigen, unlike the heat-based protocol.

## Conclusions

Our findings suggest that heat treatment, acid treatment and acid-heat treatment protocols should be considered when testing sera samples for heartworm antigen detection. In a well-characterized positive plasma diluted in a negative plasma, the heat ICD protocols, rather than acid treatment, consistently enhance the *D. immitis* antigen detection. In terms of required volume of canine sera or plasma, we favor the protocol described by Weil et al., 1985.

## Data Availability

The datasets generated during the current study are available from the corresponding author on reasonable request.

## Abbreviations

DENV: Dengue virus
DTPA: Diethylenetriaminepentaacetic acid
EDTA: Ethylenediaminetetraacetic acid
HIV: Human immunodeficiency virus
ICD: Immune complex dissociation
OD: Optical density
SDS: Sodium dodecyl sulfate
TCA: Trichloroacetic acid
